# Functional brain network properties correlate with individual risk tolerance in young adults

**DOI:** 10.1016/j.heliyon.2024.e35873

**Published:** 2024-08-06

**Authors:** Wi Hoon Jung

**Affiliations:** Department of Psychology, Gachon University, 1342 Seongnam-daero, Seongnam, 13120, Gyeonggi-do, South Korea

**Keywords:** Betweenness centrality, Graph theoretical analysis, Individual differences, Risk attitude, Risk tolerance, Small-worldness

## Abstract

**Background:**

Individuals differ substantially in their degree of acceptance of risks, referred to as risk tolerance, and these differences are associated with real-life outcomes such as risky health-related behaviors. While previous studies have identified brain regions that are functionally associated with individual risk tolerance, little is known about the relationship between individual risk tolerance and whole-brain functional organization.

**Methods:**

This study investigated whether the topological properties of individual functional brain networks in healthy young adults (n = 67) are associated with individual risk tolerance using resting-state fMRI data in conjunction with a graph theoretical analysis approach.

**Results:**

The analysis revealed that individual risk tolerance was positively associated with global topological properties, including the normalized clustering coefficient and small-worldness, which represent the degree of information segregation and the balance between information segregation and integration in a network, respectively. Additionally, individuals with higher risk tolerance exhibited greater centrality in the ventromedial prefrontal cortex (vmPFC), which is associated with the subjective value of the available options.

**Conclusion:**

These results extend our understanding of how individual differences in risk tolerance, especially in young adults, are associated with functional brain organization, particularly regarding the balance between segregation and integration in functional networks, and highlight the important role of the connections between the vmPFC and the rest of the brain in the functional networks in relation to risk tolerance.

## 1 Introduction

From birth to death, we face countless choices every day, such as choosing what to eat at each mealtime. Such choices are made by comparing the benefits of a reward with the costs of obtaining it between available alternatives. For example, whereas drinking alcohol provides a reward in the form of pleasure, it may also be detrimental to our health. As in this example, an individual that is confronted with a choice experiences uncertainty regarding which of several possible outcomes will actually occur. In particular, uncertainty about the outcome when the probability of each possible outcome is known, such as in the cases of a fair coin toss or the roulette wheel, is referred to as “risk” [1].

Individuals vary in the degree of willingness to tolerate risk, a phenomenon known as “risk tolerance” or “risk preference”. Individual differences in risk tolerance can be measured by assessing preferences between smaller-but-certain and larger-but-uncertain rewards [[2], [3], [4], [5]]. Considering previous findings showing that individual differences in risk tolerance are associated with a wide array of real-life decisions, including drinking, smoking, financial investments, and insurance coverage [6,7], as well as with susceptibility to mental illness [[8], [9], [10]], investigating the neural mechanisms underlying individual differences in risk tolerance may be useful to predict the occurrence of risky behaviors and provide clues for developing effective interventions to reduce such behaviors. Therefore, I examined the neural mechanisms underlying individual differences in risk tolerance using resting-state functional MRI (rs-fMRI) in conjunction with a graph theoretical analysis (GTA) approach.

Accumulating evidence from neuroimaging studies has identified several brain areas in which differences may be associated with differential risk tolerance [4,[11], [12], [13], [14], [15], [16], [17], [18]]. First, differences may be expected in areas representing the subjective value of the available options, including the ventral striatum, ventromedial prefrontal cortex (vmPFC), and posterior cingulate cortex [3,11,[16], [17], [18], [19], [20]]. These areas belong to parts of the valuation network, overlapping with the default mode network (DMN) associated with self-referential processing [21,22]. Second, differences may also be expected in areas associated with the salience of the stimuli, including the anterior insula and anterior cingulate cortex [[16], [17], [18], [19],[23], [24], [25]]. Third, one may also expect to find differences in areas associated with self-control processing, including the lateral prefrontal and posterior parietal cortices [16,23,26,27]. While the aforementioned regions have shown increased activation in response to increased risk across participants [20,23,26,28], the value-related activation patterns of the lateral and medial prefrontal cortices were shown to be modulated by individual risk tolerance [3,29]. Finally, differences may be expected in areas associated with emotional processing such as anxiety, including the amygdala [15,30]. Recently, my colleagues and I reported the involvement of the amygdala, and its functional and structural connectivity with the vmPFC, on individual differences in risk tolerance [15].

GTA is used to investigate the topological characteristics of large-scale functional brain networks, constructed from region-to-region functional connectivity (FC) [31,32]. In other words, GTA measures the degree of information segregation (represented by the index known as “clustering coefficient” and the “local efficiency” index related to it) and integration (represented by the “characteristic path length” and the “global efficiency” inversely related to it) of all regions as a whole in addition to the balance between segregation and integration (represented by the “small-worldness” index) in a global level [33,34]. Moreover, it also evaluates the role of a given area within the whole brain network as a hub at the regional level through an index known as the “betweenness centrality” (BC) [35], which measures the node's role in acting as a bridge between brain regions. Although few studies are available in the literature at present, previous reports using GTA have suggested the association between individual differences in decision-making (i.e., choice behaviors) and global as well as regional topological properties in brain networks [[36], [37], [38]]. For example, individual differences in behaviors related to intertemporal choice (represented by an index known as “delay discounting rate”) were associated with the small-worldness metric [36]. However, to the best of my knowledge, no studies so far have explicitly investigated whether there are associations between individual differences in risk tolerance and topological characteristics of functional brain networks.

Therefore, I investigated whether individual differences in risk tolerance in healthy young adults are associated with global and local topological properties of a functional brain network constructed using whole-brain FC. To this end, several global network metrics representing information segregation (including the clustering coefficient and local efficiency), information integration (including the characteristic path length and global efficiency), and the balance between them (including small-worldness) were measured. I also measured a regional network metric (i.e., BC) that captures the extent to which a given node lies between others. Based on previous studies relevant to risk tolerance and decision-making [11,14,36,37], I hypothesized that individual differences in risk tolerance may be associated with differences in small-worldness and BC in areas belonging to the above-mentioned functional networks, particularly in the prefrontal areas modulated by individual differences [3,29].

## 2 Materials and methods

### 2.1 Participants

Seventy-three healthy young participants were included in this study. They were recruited via online advertisements and completed both a risk tolerance task and brain scans. Six of these individuals were excluded because of (i) poor quality of the rs-fMRI data (n = 2), and (ii) being outliers (>3 standard deviations [SD] from the group mean) in the degree of risk tolerance (n = 4). People with extreme decision preferences, at floor or ceiling on the assessment (*a* < 0.34 or *a* > 1.32), were not included in the study [15]. Therefore, 67 participants were included in the final analysis (30 women and 37 men; age [mean ± SD], 24.0 ± 1.4 years; duration of education, 16.50 ± 0.71 years; risk tolerance, 0.595 ± 0.251). All participants provided written informed consent before enrollment. All the procedures were approved by the Institutional Review Board of Gachon University.

### 2.2 Risk tolerance task

**Fig. 1:**
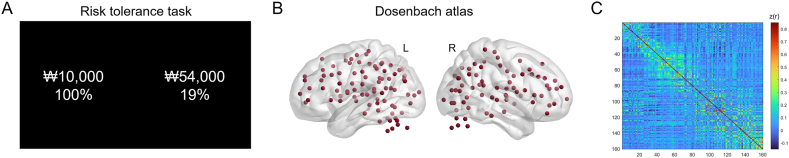
Risk tolerance task and the atlas used for the network matrix. (A) Example trial of the experimental task. Participants were asked to choose between a small but certain reward (100 % chance of KRW 10,000) and a larger but risky reward (e.g., 19 % chance of KRW 54,000). (B) The atlas with 160 areas functionally defined by Dosenbach et al. [38] was used to define nodes for the functional brain network. (C) A weighted matrix was created by averaging Fisher r-to-z-transformed functional connectivities across all participants for visualization purposes only. The color bar indicates the correlation coefficient between nodes.

On each trial, participants were asked to choose between a smaller reward available with certainty and a larger reward available with some risk (Fig. 1A). The smaller but certain reward was fixed at a 100 % chance of 10,000 Korean won (KRW, approximately USD 8–9) and the larger but risky rewards varied according to the individual trial. The magnitude of the larger but risky rewards ranged from KRW 11,000 to KRW 63,000, and the probability of obtaining it varied from 13 % to 98 %. Each participant completed a total of 120 choices during the task. There was no time limit for making a choice, but participants were instructed to make their choice as quickly as possible when presented with options. At the end of the experiment, participants received the item they chose on one randomly selected trial from the task, in addition to visit compensation. The gamble in the trial randomly chosen was resolved by the roll of a die [15,18]. Individual behavioral data were fitted using a logistic regression function with a maximum likelihood estimate in order to capture the probability of choosing the larger but risky reward as a stochastic function of the difference in subjective value (SV) between the two options. To estimate risk tolerance, the SV was assumed to follow the function form of expected utility, a power-law function of the reward amount (*A*), and the probability (*P*) of winning: SV = *P* × *A*^*a*^, where *a* corresponds to the participant's degree of risk tolerance. A detailed description of the risk tolerance estimation method can be found elsewhere [15,18,39]. The *a* parameter was log-transformed to normalize the distribution before statistical analyses (log-transformed *a* [mean ± SD], −0.267 ± 0.198). A Shapiro–Wilk test was performed and showed a normal distribution of the log-transformed *a* value (W = 0.965; p = 0.057). Larger values of *a* indicate a greater degree of risk tolerance or a lesser degree of risk sensitivity; that is, *a* values above 1 represent risk seeking, whereas values below or equal to 1 represent risk aversion or neutrality, respectively.

### 2.3 Image acquisition

All imaging data were collected using a 3T Trio MRI scanner (Siemens, Erlangen, Germany). High-resolution T1-weighted anatomical images were obtained using a 3D magnetization-prepared rapid-gradient echo (MPRAGE) sequence (repetition time [TR] = 1900 ms, echo time [TE] = 2.52 ms, flip angle [FA] = 9°, voxel size = 1.0 × 1.0 × 1.0 mm^3^, 192 sagittal slices). rs-fMRI data were acquired using T2*-weighted echo-planar imaging (TR = 2000 ms, TE = 20 ms, FA = 90°, voxel size = 3.0 × 3.0 × 3.0 mm^3^, 45 interleaved axial slices, and 155 volumes). During the scans, the participants were instructed to keep their eyes open and maintain fixation. An eye tracker mounted on a head coil was used to monitor their eyes and ensure that they did not fall asleep during the scanning procedure.

### 2.4 Imaging preprocessing

Image data were preprocessed using the Data Processing Assistant for Resting-State fMRI Advanced Edition (DPARSFA) toolbox (www.rsfMRI.org/DPARSF) [40] with SPM12 (www.fil.ion.ucl.ac.uk/spm). Preprocessing steps included the removal of the first four volumes, slice-acquisition timing, motion correction, nuisance signal regression, spatial normalization, spatial smoothing, and temporal filtering. All data showed (i) six motion parameters <1 voxel (2.5 mm or 2.5°) in any direction and rotation, and (ii) a mean frame-wise displacement (FD) < 0.30 [41]. To remove the effect of head motion and non-neuronal fluctuations on signals, the following nuisance parameters were included as regressors within the general linear model: Friston 24-motion parameters, five principal components estimated from the individual white matter and cerebrospinal fluid mask using an anatomical component-based noise correction method [42], and a linear de-trending term. Next, the residual images were normalized to the Montreal Neurological Institute (MNI) space and then smoothed with a 6 mm full-width half-maximum Gaussian kernel. Finally, time series were band-pass filtered (0.01–0.1 Hz).

### 2.5 Graph theoretical analysis

Network construction and analyses were performed using the Graph Theoretical Network Analysis (GRETNA) toolbox (https://www.nitrc.org/projects/gretna) [43]. An atlas with 160 regions functionally defined by Dosenbach et al. [44] was used to determine nodes in the network (Fig. 1B). The regions included in the Dosenach atlas (i.e., 160 nodes; Supplementary Table 1) were identified from meta-analyses of task-related fMRI studies. This atlas has been widely used for GTA of functional brain networks [45,46]. The strengths of FC as edges were measured by computing the Pearson correlation coefficient for every pair of regional time series, generating a 160 × 160 weighted network for each participant (Fig. 1C). Given the absence of a gold standard for selecting a single threshold for the constructed correlation matrix, the matrix was binarized based on only positive correlations (i.e., negative connections were set to 0) using a wide range of sparsity thresholds *S* (0.10 < *S* < 0.40 with an interval of 0.01). This *S* range was selected to allow for prominent small-world properties in the brain networks of all participants; that is, the small-worldness (*σ*) in the defined sparsity range was greater than 1 [33].

Several global topological properties were estimated, including small-world parameters (i.e., clustering coefficient [*C*_*p*_], characteristic path length [*L*_*p*_], small-worldness (*σ*), and global [*E*_*glob*_] and local [*E*_*loc*_] efficiency), and a regional topological property, including BC. The equations used to estimate all these metrics can be found elsewhere [31,47,48]. Briefly, in the global level, the *C*_*p*_ is defined as the average of how densely the neighbors of each node in a network are connected [33], and the *E*_*loc*_ of a network, related to the *C*_*p*_, is defined as the average of the local efficiencies of all nodes [49]. The local efficiency of a node represents a quantification of how well information is exchanged by its neighbors when it is removed. Both the *C*_*p*_ and *E*_*loc*_ are related to network segregation. The *L*_*p*_ of a network is defined as the average shortest path length between all node pairs in the network [50] and the *E*_*glob*_ is inversely related to the *L*_*p*_ [34]. Both *L*_*p*_ and *E*_*glob*_ are related to network integration. Small-worldness is defined as the ratio between normalized *C*_*p*_ and normalized *L*_*p*_, by comparing 100 matched random networks [51]. In other words, before computing the ratio, the *C*_*p*_ and *L*_*p*_ were normalized by comparing them to the corresponding mean values of 100 matched random networks. BC is defined as the fraction of all shortest paths in a network running through a given node [35]. Before the statistical analyses, the BC for each node was normalized by dividing their average values of the network.

### 2.6 Statistical analysis

The area under the curve (AUC) was estimated for each topological metric to investigate its association with risk tolerance and to avoid arbitrariness in threshold selection. Before computation, multiple linear regression analyses were conducted to remove the confounding effects of age, sex, and education level (years). Spearman's rank correlation analysis was then performed to examine the associations between individual risk tolerance (log-transformed *a*) and each of the network topological properties. The level of statistical significance was set at p < 0.05. For BC as the regional property, I corrected the significance for the number of nodes using a less stringent false positive adjustment [i.e., p < (1/160) = 0.006], following previous studies [[52], [53], [54], [55], [56], [57]].

## 3 Results

### 3.1 Global network measures

**Fig. 2:**
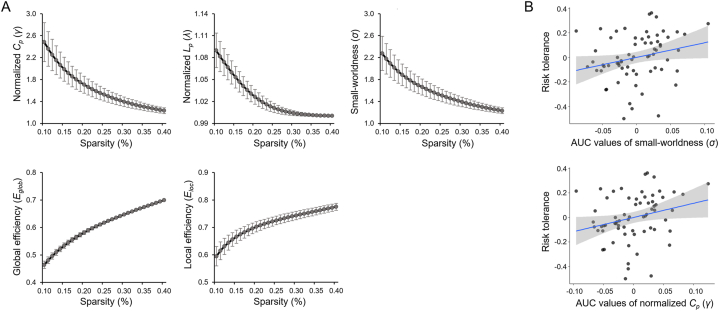
Global topological properties of the functional brain network and their association with risk tolerance. (A) The global measures included the normalized clustering coefficient, the normalized characteristic path length, small-worldness, global efficiency, and local efficiency. For all participants, a small-world network architecture (γ > 1, *λ* ≈ 1, and *σ >* 1) was observed over the defined sparsity range (0.10 < *S* < 0.40) (B) Individual risk tolerance (i.e., log-transformed *a*) was positively correlated with the area under the curve (AUC) values of small-worldness and the normalized clustering coefficient. For illustration purposes, correlation scatterplots were generated by performing Pearson correlation analysis between residuals after regressing out age, sex, and education. *C*_*p*_, clustering coefficient; *L*_*p*_, characteristic path length.

All participants showed a small-world architecture (normalized *C*_*p*_ [γ] > 1, normalized *L*_*p*_ [*λ*] ≈ 1, and small-worldness [*σ*] *>* 1; Fig. 2A) in the whole-brain functional network within the defined sparsity range.

Individual risk tolerance was positively correlated with the AUCs of small-worldness (r-/p-values = 0.273/0.026) and of the normalized *C*_*p*_ (r-/p-values = 0.288/0.018), indicating that higher risk tolerances were associated with higher values in the AUCs of small-worldness and of normalized *C*_*p*_, respectively (Fig. 2B). Other global properties were not associated with individual risk tolerance (*λ*, r-/p-values = 0.175/0.156; *E*_*glob*_, −0.164/0.1843; *E*_*loc*_, 0.200/0.105).

### 3.2 Regional network measures

**Fig. 3:**
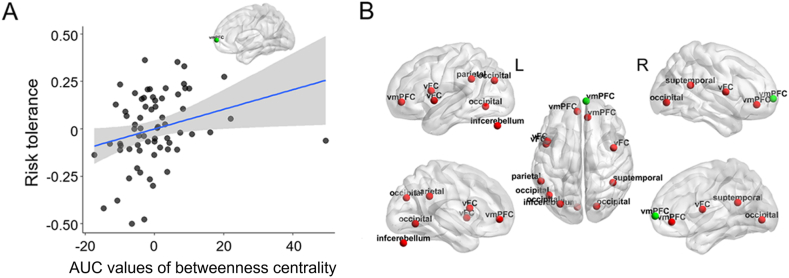
Associations between risk tolerance and betweenness centrality (BC) as a regional topological metric. (A) Individual risk tolerance was positively associated with the AUC values of BC of the ventromedial prefrontal cortex (vmPFC) at p < 0.006 (=1/160) for correction based on the number of nodes. (B) Application of a less stringent threshold (p < 0.05) to explore trends showed some additional correlations between individual differences in risk tolerance and the regional topological properties of several nodes located in the frontal, parietal, and temporal areas. Red circles on the brain images represent regions showing correlations between risk tolerance and the AUCs of BC at p < 0.05. Green circles indicate the vmPFC node showing a significant correlation with risk tolerance at p < 0.006. Detailed information for these regions (i.e., coordinates) is provided in Table 1 vmPFC, ventromedial prefrontal cortex; vFC, ventral frontal cortex; intracerebellum, inferior cerebellum; suptemporal, superior temporal cortex.

Individual risk tolerance was significantly correlated only with the AUC value of BC of the right vmPFC (x, y, z coordinates = 6, 64, 3; r-/p-values = 0.354/0.003; Fig. 3A). Application of a less stringent significance threshold in order to determine trends due to the exploratory nature of this analysis with mass-univariate correlation yielded additional correlations between risk tolerance and the BC of several nodes located in the ventral and dorsal part of the frontal cortex and in occipital and parietal areas (p < 0.05; Fig. 3B). The regions that showed the highest correlation coefficients (top 10 %) between risk tolerance and AUCs of BC are listed in Table 1.

**Table 1:** Regions with the highest correlation coefficients (top 10 %) between risk tolerance and betweenness centrality (BC).

#Rank	Area	MNI coordinates			Correlation with *BC*	
		x	y	z	r-value	p-value
1	**vmPFC**	**6**	**64**	**3**	**0.354**	**0.003**^a^
2	**vmPFC**	**8**	**42**	**−5**	**0.297**	**0.015**
3	**vFC**	**−48**	**6**	**1**	**−0.281**	**0.021**
4	**vmPFC**	**−6**	**50**	**−1**	**0.280**	**0.022**
5	**occipital**	**−44**	**−63**	**−7**	**−0.277**	**0.023**
6	**inf cerebellum**	**−6**	**−79**	**−33**	**0.277**	**0.024**
7	**occipital**	**−29**	**−75**	**28**	**0.275**	**0.025**
8	**parietal**	**−55**	**−44**	**30**	**−0.270**	**0.027**
9	**vFC**	**−46**	**10**	**14**	**−0.253**	**0.039**
10	**sup temporal**	**42**	**−46**	**21**	**0.244**	**0.047**
11	**occipital**	**20**	**−78**	**−2**	**0.244**	**0.047**
12	**vFC**	**43**	**1**	**12**	**0.243**	**0.048**
13	occipital	45	−72	29	0.228	0.064
14	post cingulate	−11	−58	17	−0.227	0.065
15	dFC	40	17	40	−0.224	0.069
16	inf temporal	52	−15	−13	0.222	0.071

Brain areas with p < 0.05 for the correlation with the AUC values of BC are shown in bold. vmPFC, ventromedial prefrontal cortex; vFC, ventral frontal cortex; inf, inferior; sup, superior; dFC, dorsal frontal cortex; MNI, Montreal Neurological Institute.

a p < 0.006 (1/160) for multiple comparison correction based on the number of nodes.

### 3.3 Supplementary analyses

The scatter plot for the correlation between the AUC of BC of the right vmPFC and risk tolerance (Fig. 3A) suggests that the result may be due to the presence of one outlier on the values of the vmPFC node (>3 SD from the mean). Therefore, the above-mentioned correlation analysis was repeated after exclusion of the outlier data point, but the significant associations that had been initially observed were still present (Supplementary Fig. 1). Individual risk tolerance was positively associated with the AUCs of small-worldness and normalized *C*_*p*_ (r-/p-values = 0.271/0.028 and 0.285/0.021, respectively). An association between individual differences in risk tolerance and the AUC of the vmPFC BC was also observed (r-/p = 0.372/0.002). The AUCs of BC corresponding to other brain areas showed no significant correlation with individual risk tolerance (p > 0.006 in all cases).

## 4 Discussion

This study explicitly investigated the associations between individual differences in risk tolerance and the global and regional topological properties of whole-brain functional networks, constructed by regional-to-regional functional couplings, in healthy young adults. The current study showed an association between individual differences in risk tolerance and the global topological properties of functional brain networks, including normalized *C*_*p*_ and small-worldness, indicating that individuals with higher risk tolerance have an increase in information segregation and in the balance between segregation and integration in the networks. Additionally, individual differences in risk tolerance were linked to differences in the BC of the right vmPFC, an area associated with the subjective value assigned to the available options. While previous studies focused on risk tolerance and risk behavior have provided evidence on the association of risk tolerance with specific brain networks (such as valuation and salience networks) or on the involvement of the bivariate FC between vmPFC and other regions in risk tolerance, the current study further extends our knowledge on this phenomenon by providing the novel findings mentioned above, which suggest that whole-brain functional organization patterns at the global and regional levels underlie individual differences in risk tolerance.

In a brain network, information is processed by being integrated and separated between regions. A small-world network is considered to have an optimal balance between information segregation and integration [33]. In other words, a small-world network is considered the most efficient model, allowing efficient communication over both short and long distances, and is characterized by a high *C*_*p*_ (similar to a regular network) and a short *L*_*p*_ (similar to a random network) [33]. Among various global topological metrics, small-world parameters (including *C*_*p*_, *L*_*p*_, *E*_*glob*_, *E*_*loc*_ and *σ*) measure such segregation (*C*_*p*_ and *E*_*loc*_) and integration (*L*_*p*_ and *E*_*glob*_) of the information, as well as the balance between them (small-worldness, *σ*) [[33], [34], [35],[49], [50], [51]].

In the current study, the architecture features of the functional brain networks from each participant met the criteria for a small-world network (i.e., normalized *C*_*p*_ [γ] > 1, normalized *L*_*p*_ [*λ*] ≈ 1, and small-worldness [*σ*] *>* 1) in the defined sparsity range, which is consistent with the results of previous studies [31,32,48,58]. Individual differences in risk tolerance were also positively associated with those in small-worldness, indicating that individuals with higher risk tolerance had greater small-worldness. One possible interpretation is that this may reflect the important role of efficient communication between the mentioned brain areas (including the ventral striatum, vmPFC, posterior cingulate cortex, anterior insula, anterior cingulate cortex, posterior parietal cortex, lateral and medial prefrontal cortices, and amygdala; see Introduction) on risk tolerance [3,11,[15], [16], [17], [18], [19], [20], [21], [22],^26^]. That is, based on the functional role of these areas, the results suggest that communication of information relevant to the estimation of value and salience of the stimuli, to self-control, and to emotional processing is important in the determination of risk tolerance. Although individuals with higher risk tolerance showed greater small-worldness, I caution against this interpretation, as all the participants were healthy young adults who did not present any clinical problems. Based on the equation for small-worldness (i.e., the ratio between normalized *C*_*p*_ and normalized *L*_*p*_) [33], another possible interpretation is that the greater the difference between *C*_*p*_ and *L*_*p*_, the higher the risk tolerance. In other words, a higher small-worldness can occur if the *C*_*p*_ (the numerator) is relatively greater than *L*_*p*_ (the denominator). Indeed, in the current study, a higher *C*_*p*_ was associated with higher risk tolerance, indicating a positive relationship between individual differences in risk tolerance and *C*_*p*_. A high *C*_*p*_ results from a high number of connections between neighboring rather than remote regions relative to the total number of possible connections, and is also known as a good indicator of the robustness of a network, defined as the ability of the network to resist damage [59]. This suggests that individuals with a higher risk tolerance have more connections between neighboring brain regions.

Based on previous research findings [[60], [61], [62]], it was inferred that there would be a relationship between risk tolerance and the network topology of the brain network. For example, alterations in small-world properties have been observed among people who engage in risky behaviors such as addiction [60,61], although results are inconsistent across studies [62,63]. In addition, Luo et al. [64] recently observed that small-world properties of the resting-state functional brain network are linked to drug use, psychological craving, and impulsivity. Therefore, based on previous results in clinical patients and the current findings with healthy young adults, it is suggested that risk tolerance is related to global topological properties of functional brain networks.

This study examined graph measures for functional networks, not structural networks. Functional connections do not represent physical pathways that could facilitate communication or information transfer between regions. Rather, they capture statistical associations between regional time courses (i.e., the covariations of the activity of two regions), which can be considered as the outcome of information integration via structural connections. In other words, a functional connection is not a direct flow of information along a specific path, but rather a collection of different information flows through multiple paths [65]. As such, there is debate regarding the above-mentioned interpretation that the graph measures capture information integration in FC networks. Despite these considerations, it is certain that functional connections convey the patterns of information flow through underlying structural connections [65], even if they (functional and structural connections) are not perfectly matched. Future research using structural connectivity data (e.g., diffusion tensor imaging data) should be performed to determine whether the similar results are obtained in structural networks.

In the current study, individual differences in risk tolerance were positively associated with the BC of the right vmPFC, that is, the FCs between the vmPFC and the rest of the brain. The vmPFC is related to decision-making, in particular with the encoding of the subjective values for the available options [11,15], as well as with risk-taking behaviors [14,66]. A previous investigation suggested that damage to the vmPFC can lead to problems in learning to value stimuli with uncertain incentives (rewards or punishments) due to deficient learning from feedback [e.g., overestimation (underestimation) of the positive (negative) value of potential gains (losses)] [14]. For example, patients with a lesioned vmPFC had a deficient performance on the Iowa gambling task [66]. Therefore, the current findings showing a positive association between differences in risk tolerance and the BC of the vmPFC may reflect inappropriate feedback processing. In other words, individuals with higher risk tolerance may engage in risky behaviors because they overestimate the potential value of potential gains and/or underestimate the negative value of potential losses. The vmPFC is connected to various cortical and subcortical areas, and these connections play a crucial role in the regulation of risk tolerance [15,67,68]. For example, colleagues and I have recently observed an association between individual differences in risk tolerance and the vmPFC–amygdala functional and structural connectivity [15]. More recently, Wang et al. [67] reported a positive correlation between changes in risk-taking and the FC between the vmPFC and the dorsolateral PFC before and after sleep deprivation. Rolls et al. [68] compared 4891 risk-taking individuals with 13,849 non-risk-taking individuals and reported higher FC in multiple areas (including the vmPFC) in the risk-taking group. Taken together, these results suggest that altered FC in the vmPFC may cause risky behaviors.

Graph measures used in the current study (characteristic path length, small-worldness, and BC) assume that information integration occurs exclusively via the shortest paths between regions. However, there is growing empirical evidence against this assumption [65,69]. For example, the idea that neural signals flow throughout the shortest paths in the network implies that neural signals can access to knowledge of the global network topology [65]. This assumption is considered highly unlikely in physiological system. As such, alternative graph measures, which are not based entirely on the shortest path structure of the network, may serve as more accurate description of integration processes in the brain [65,69]. For instance, Grayson et al. [69] pharmacogenetically inactivated the amygdala of the rhesus money and investigated the impact of that perturbation on functional brain networks. Especially, the study applied a specific graph measure, called “communicability”, to identify the sites of inactivation that best explain functional effects. The communicability makes no assumptions about the priority of the shortest path, allowing contributions from all paths of reasonably short length [70]. The study showed a stronger correspondence between communicability and FC when incorporating the directionality of structural connections [69].

Several possible limitations should be considered. First, the current study only focused on young, healthy college students. This restricted range may limit the generalizability of the findings. Considering the available evidence for age-related changes in risk tolerance and their associated neural mechanisms [13], further studies with diverse age ranges are needed to clarify whether age-related changes exist in the relationship between risk tolerance and network topological properties. Second, considering the vast number of possible connections (160 × 159/2 = 12,720) in the defined brain network, the final sample size (n = 67) may have led to low statistical power, which may affect the generalizability of the findings. That is, results obtained from relatively small sample sizes may raise concerns about the reliability and reproducibility. Therefore, future studies with larger samples are needed to verify the current findings. Third, risk tolerance has been reported to be related to an individual's demographic characteristics, including age and sex [13,71], and cognitive abilities, including specific cognitive function (e.g., working memory capacity) and general intelligence (i.e., IQ) [[72], [73], [74]]. Therefore, since it is important to control potential confounding variables related to risk tolerance, multiple linear regression analysis was conducted to remove the confounding effects of age, sex, and education. However, in this study, IQ was not obtained and thus could not be controlled. Fourth, based on previous studies [[52], [53], [54], [55], [56], [57]], a less stringent multiple comparison correction (p < 1/160) was used to investigate the association of global topological features with risk tolerance. Liberal statistical thresholds do not guarantee against false positives but provide more information about the association between variables. Finally, when constructing a network in GTA, a binary or weighted matrix can be used. Both binary and weighted matrices have their advantages and disadvantages. For example, the binary matrix has the advantages of simplicity and computational efficiency. In other words, it is concise and easy to understand because it only expresses whether there is a connection or not. It is also efficient for large-scale graphs because it is computationally simple and requires little memory. However, it has the disadvantage of causing information loss because it does not consider weights or relationship strengths. Unlike the binary matrix, the weighted matrix has the advantage of having detailed information but has the disadvantage of being more complex and difficult to interpret than the binary matrix. Considering the pros and cons of each matrix, additional analyses were conducted to determine whether the results obtained using the binary matrix were maintained even when the weighted matrix was used. The additional analyses with the weighted matrix revealed that individual risk tolerance has a significant positive correlation only with the AUCs of small-worldness and of the normalized *C*_*p*_, consistent with the binary matrix results (Supplementary Fig. 2). However, the significant correlation between individual risk tolerance and the AUC of vmPFC BC was not maintained. Therefore, which matrix is used may have some influence on the results.

In conclusion, this study examined whether individual risk tolerance, especially in young adults, is associated with whole-brain functional organization patterns using regional-to-regional FC in conjunction with GTA. The results revealed that individual differences in risk tolerance in young adults were associated with those in global and regional topological properties, including small-worldness, associated with the balance between information segregation and integration, and BC of the vmPFC, associated with the valuation to stimuli. These results extend our understanding of how individual differences in risk tolerance in young adults are associated with functional brain organizations in the entire brain, and suggest an important role for the FC between the vmPFC and the rest of the brain on risk evaluation, providing additional insight into the neural mechanisms underlying individual differences in risk tolerance.

## Ethics statement

This study was reviewed and approved by the Institutional Review Board of Gachon University, with the approval number: IRB#1044396-202203-HR-058-01. All participants provided informed consent to participate in the study.

## Data availability statement

Data will be made available on reasonable request.

## CRediT authorship contribution statement

**Wi Hoon Jung:** Writing – review & editing, Writing – original draft, Visualization, Supervision, Methodology, Investigation, Funding acquisition, Data curation, Conceptualization.

## Declaration of competing interest

The authors declare that they have no known competing financial interests or personal relationships that could have appeared to influence the work reported in this paper.
