# Supplementary material for: Functional brain network properties correlate with individual risk tolerance in young adults
Source: Heliyon. 2024 Aug 6;10(15):e35873. doi: 10.1016/j.heliyon.2024.e35873 (PMC11337038; doi:10.1016/j.heliyon.2024.e35873)
Supplement: Multimedia component 1 [file mmc1.pdf]

Supplementary Materials

Supplementary Table 1. The coordinates and labels of 160 nodes from Dosenbach et al. (2010)

Node#	Label	MNI coordinates			Node#	Label	MNI coordinates			Node#	Label	MNI coordinates		
		X	Y	Z			X	Y	Z			X	Y	Z
1	vmPFC	6	64	3	55	IPS	32	-59	41	109	parietal	-55	-22	38
2	mPFC	0	51	32	56	aPFC	27	49	26	110	precentral gyrus	-54	-22	22
3	aPFC	-25	51	27	57	vPFC	34	32	7	111	temporal	-54	-22	9
4	vmPFC	9	51	16	58	ACC	-2	30	27	112	parietal	41	-23	55
5	vmPFC	-6	50	-1	59	vFC	51	23	8	113	post insula	42	-24	17
6	vmPFC	-11	45	17	60	ant insula	38	21	-1	114	parietal	18	-27	62
7	vmPFC	8	42	-5	61	dACC	9	20	34	115	parietal	-38	-27	60
8	ACC	9	39	20	62	ant insula	-36	18	2	116	parietal	-24	-30	64
9	vlPFC	46	39	-15	63	basal ganglia	-6	17	34	117	post parietal	-41	-31	48
10	sup frontal	23	33	47	64	mFC	0	15	45	118	temporal	-41	-37	16
11	sup frontal	-16	29	54	65	vFC	-46	10	14	119	temporal	-53	-37	13
12	inf temporal	52	-15	-13	66	basal ganglia	-20	6	7	120	sup parietal	34	-39	65
13	inf temporal	-59	-25	-15	67	basal ganglia	14	6	7	121	occipital	-18	-50	1
14	post cingulate	1	-26	31	68	vFC	-48	6	1	122	occipital	-34	-60	-5
15	fusiform	28	-37	-15	69	mid insula	37	-2	-3	123	occipital	36	-60	-8
16	precuneus	-3	-38	45	70	thalamus	-12	-3	13	124	temporal	46	-62	5
17	post cingulate	-8	-41	3	71	thalamus	-12	-12	6	125	occipital	-44	-63	-7
18	inf temporal	-61	-41	-2	72	thalamus	11	-12	6	126	occipital	19	-66	-1
19	occipital	-28	-42	-11	73	mid insula	32	-12	2	127	occipital	17	-68	20
20	post cingulate	-5	-43	25	74	mid insula	-30	-14	1	128	occipital	39	-71	13
21	precuneus	9	-43	25	75	basal ganglia	11	-24	2	129	occipital	29	-73	29
22	precuneus	5	-50	33	76	post insula	-30	-28	9	130	occipital	-29	-75	28
23	post cingulate	-5	-52	17	77	temporal	51	-30	5	131	occipital	-16	-76	33
24	post cingulate	10	-55	17	78	post cingulate	-4	-31	-4	132	occipital	9	-76	14
25	precuneus	-6	-56	29	79	fusiform	54	-31	-18	133	occipital	15	-77	32
26	post cingulate	-11	-58	17	80	precuneus	8	-40	50	134	occipital	20	-78	-2
27	angular gyrus	51	-59	34	81	parietal	58	-41	20	135	post occipital	-5	-80	9
28	angular gyrus	-48	-63	35	82	temporal	43	-43	8	136	post occipital	29	-81	14
29	precuneus	11	-68	42	83	parietal	-55	-44	30	137	post occipital	33	-81	-2
30	IPS	-36	-69	40	84	sup temporal	42	-46	21	138	post occipital	-37	-83	-2
31	occipital	-9	-72	41	85	angular gyrus	-41	-47	29	139	post occipital	-29	-88	8
32	occipital	45	-72	29	86	temporal	-59	-47	11	140	post occipital	13	-91	2
33	occipital	-2	-75	32	87	TPJ	-52	-63	15	141	post occipital	27	-91	2
34	occipital	-42	-76	26	88	frontal	58	11	14	142	post occipital	-4	-94	12
35	aPFC	29	57	18	89	dFC	60	8	34	143	lat cerebellum	-28	-44	-25
36	aPFC	-29	57	10	90	vFC	-55	7	23	144	lat cerebellum	-24	-54	-21
37	vent aPFC	42	48	-3	91	pre-SMA	10	5	51	145	inf cerebellum	-37	-54	-37
38	vent aPFC	-43	47	2	92	vFC	43	1	12	146	lat cerebellum	-34	-57	-24
39	vlPFC	39	42	16	93	SMA	0	-1	52	147	med cerebellum	-6	-60	-15
40	dlPFC	40	36	29	94	frontal	53	-3	32	148	inf cerebellum	-25	-60	-34
41	ACC	-1	28	40	95	precentral gyrus	58	-3	17	149	inf cerebellum	32	-61	-31
42	dlPFC	46	28	31	96	mid insula	-42	-3	11	150	med cerebellum	-16	-64	-21
43	vPFC	-52	28	17	97	precentral gyrus	-44	-6	49	151	lat cerebellum	21	-64	-22
44	dlPFC	-44	27	33	98	parietal	-26	-8	54	152	med cerebellum	1	-66	-24
45	dFC	40	17	40	99	precentral gyrus	46	-8	24	153	inf cerebellum	-34	-67	-29
46	dFC	44	8	34	100	precentral gyrus	-54	-9	23	154	med cerebellum	-11	-72	-14
47	dFC	-42	7	36	101	precentral gyrus	44	-11	38	155	inf cerebellum	33	-73	-30
48	IPL	-41	-40	42	102	parietal	-47	-12	36	156	med cerebellum	5	-75	-11
49	IPL	54	-44	43	103	mid insula	33	-12	16	157	med cerebellum	14	-75	-21
50	post parietal	-35	-46	48	104	mid insula	-36	-12	15	158	inf cerebellum	-21	-79	-33
51	IPL	-48	-47	49	105	temporal	59	-13	8	159	inf cerebellum	-6	-79	-33
52	IPL	-53	-50	39	106	parietal	-38	-15	59	160	inf cerebellum	18	-81	-33
53	IPL	44	-52	47	107	parietal	-47	-18	50					
54	IPS	-32	-58	46	108	parietal	46	-20	45					

Supplementary Materials

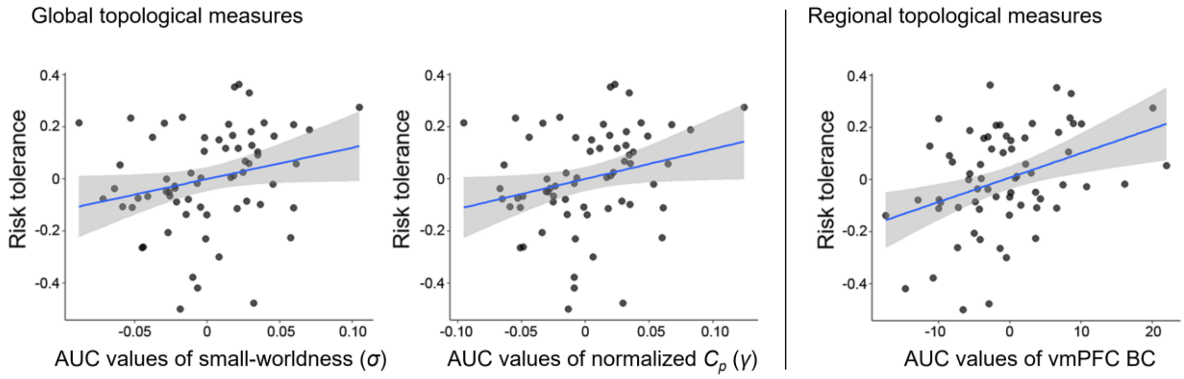

Supplementary Figure 1. Results from supplementary analyses showing associations between risk tolerance and network topological properties. The data was re-analyzed after subtraction of one outlier data point [defined by betweenness centrality (BC) > 3 standard deviations above the mean] in order to test whether the findings previously observed remained significant. The re-analysis revealed that the associations between risk tolerance and the area under the curve (AUC) of small-worldness (r-/p-values = 0.27/0.03), of normalized C_p (r-/p-values = 0.29/0.02), and of the BC of the ventromedial prefrontal cortex (vmPFC) (r-/p-values = 0.372/0.002; x, y, z coordinates = 6, 64, 3) remained significant. For illustration purposes, these correlation scatterplots were generated by performing Pearson correlation analysis between residuals after regressing out age, sex, and education.

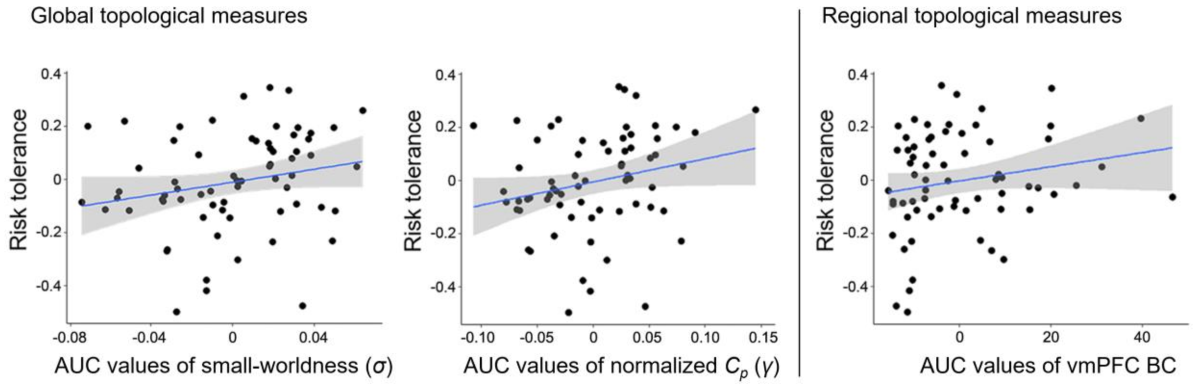

Supplementary Figure 2. Results for the weighted network. Additional analyses were conducted to determine whether the results obtained using the binary matrix were maintained even when the weighted matrix was used. The additional analyses with the weighted matrix revealed that individual risk tolerance has a significant positive correlation only with the AUCs of small-worldness (r-/p-values = 0.25/0.04) and of the normalized C_p (r-/p-values = 0.25/0.04), consistent with the binary matrix results. However, the correlations between individual risk tolerance and the betweenness centrality (BC) values of ventromedial prefrontal cortex (vmPFC) and of other brain areas were not significant (p > 0.006 in all cases). For illustration purposes, these correlation scatterplots were generated by performing Pearson correlation analysis between residuals after regressing out age, sex, and education.

Supplementary Materials

References

N.U. Dosenbach, B. Nardos, A.L. Cohen, D.A. Fair, J.D. Power, J.A. Church, et al., Prediction of individual brain maturity using fMRI, Science 329 (2010) 1358-1361.
